# Laboratory and clinical features of tumor lysis syndrome in children with non-Hodgkin lymphoma and evaluation of long-term renal functions in survivors

**DOI:** 10.1186/s12887-024-04549-w

**Published:** 2024-01-31

**Authors:** Selcen Bozkurt, Dildar Bahar Genc, Sema Vural

**Affiliations:** 1 Department of Pediatric Allergy-Immunology, Marmara University School of Medicine, Istanbul, Turkey; 2 Department of Pediatric Oncology, University of Health Sciences, Sisli Hamidiye Etfal Training and Research Hospital, Istanbul, Turkey

**Keywords:** Acute kidney injury, Chronic kidney disease, Non-Hodgkin lymphoma, Tumor lysis syndrome

## Abstract

**Objective:**

The purpose of our study is to investigate the laboratory and clinical features of tumor lysis syndrome (TLS) and acute kidney injury (AKI) in childhood non-Hodgkin lymphomas (NHL) and to reveal their impact on long term kidney function in survivors.

**Methods:**

Our single-center retrospective study included 107 patients (0-18 years old) with NHL who were admitted and treated at our hospital between 1998 and 2020. The relationship between TLS and age, gender, histopathological subgroup, tumor stage, lactate dehydrogenase (LDH) level at presentation, bone marrow and kidney involvement were assessed. The long-term renal functions of the patients were investigated.

**Results:**

80.3% of the patients were male with a median age of 9.8 years. The most common detected histopathological subgroup was Burkitt lymphoma. Hyperhydration with or without alkalinisation, and allopurinol were used in first-line treatment and prophylaxis of TLS. Laboratory TLS and clinical TLS was observed in 30.8% and 12.1% of patients, respectively. A significant correlation was found between young age, advanced stage, high LDH level at presentation, and TLS. AKI was observed in 12.1% of the patients. When the glomerular filtration rate values of the patients at the first and last admissions were compared after an average of 6.9 years, a mean decrease of 10 mL/min/1.73 m2 was found. It was not, however, found to be statistically significant.

**Conclusion:**

Lower age, advanced stage, and high LDH level at presentation were found to be risk factors for TLS in our study. Long-term renal function loss was not observed in the survivors who received early and careful prophylaxis/treatment for TLS. The survivors are still being followed up.

## Introduction

Tumor lysis syndrome (TLS) refers to biochemical and clinical abnormalities caused by spontaneous or treatment-induced necrosis of rapidly proliferating large tumor [1]. Laboratory TLS (LTLS) is defined by Cairo and Bishop as two or more the following biochemical abnormalities (beyond the normal limit or 25% change from baseline) occurring three days before or within seven days of starting chemotherapy: elevated serum uric acid, potassium, and phosphate levels, or decreased serum calcium levels. Clinical tumor lysis syndrome (CTLS) is diagnosed if, along with LTLS, there is a serum creatinine level exceeding 1.5 times the upper limit of normal range, or a cardiac arrhythmia/sudden death or a seizure [1, 2].

The incidence of TLS ranging from 4.4% to 53.6% has been reported in studies conducted on hematologic malignancies in children [3]. Its incidence can vary depending on factors such as tumor type, individual differences, and treatment plans. It is frequently observed in rapidly proliferating hematologic malignancies such as acute leukemia and non-Hodgkin lymphomas (NHL), while being less commonly observed in solid tumors [4–6]. In TLS, in addition to high proliferation rate, high tumor sensitivity to chemotherapy, advanced stage (stage III-IV), high serum lactate dehydrogenase (LDH) levels, bone marrow involvement and kidney involvement are well-known risk factors [1–4].

Tumors exhibiting rapid proliferation release intracellular contents such as anions, cations, nucleic acid breakdown products, and cytokines into circulation due to necrosis induced by spontaneous or cytotoxic treatment. Elevated levels of serum phosphorus, uric acid, and potassium disrupt the normal hemostatic mechanisms. When the secretion capacity of kidney tubules is exceeded, TLS occurs [1, 4, 7]. The most crucial step in the management of TLS is preventing the occurrence of TLS. Prophylactic management involves ensuring adequate hydration and the use of hypouricemic agents such as allopurinol and rasburicase [1–4].

Acute kidney injury (AKI) is a serious complication of TLS [8]. In TLS, the accumulation of calcium phosphate crystals and uric acid crystals in renal tubules due to hyperuricemia and hyperphosphatemia plays a significant role in the pathogenesis of AKI. Slightly elevated uric acid levels can also lead to the development of AKI through inducing renal vasoconstriction and local inflammation although they do not directly cause tubular obstruction [2, 8, 9].

AKI may develop due to dehydration, use of nephrotoxic agents and disease involvement without uric acid nephropathy [3, 10]. Toxic agents can disrupt renal tubular secretion and reabsorption by causing damage to metabolically active kidney cells. Ifosfamide and cisplatin are known as the two chemotherapeutic agents that most commonly induce nephrotoxicity. While rarely observed in children, these chemotherapeutic agents can also lead to acute nephrotoxicity. Manifestations of tubulopathy are more commonly seen in the subacute and chronic phases. When acute nephrotoxicity occurs, the use of the chemotherapeutic agent responsible for this condition should be discontinued [11, 12]. In renal involvement, the tumor itself causes damage to renal tissue or disrupts renal functions by causing obstructive uropathy, leading to AKI [3].

AKI may accompany CTLS. However, not all AKI is CTLS. AKI may develop due to dehydration, use of nephrotoxic agents and disease involvement without uric acid nephropathy. The renal criterion for CTLS is defined as a 1.5 times increase in creatinine above the upper limit of normal values for the patient’s age and gender, which does not always overlap with the AKI criteria, namely an increase in baseline creatinine or a decrease in urine output.

AKI is not a self-limiting condition. Tubulointerstitial fibrosis can be caused by long-term interstitial inflammation and chronic hypoxia. As a result of progressive nephron loss, hypertension may develop and cause a decrease in glomerular filtration rate (GFR) [10, 13–15].

Recent studies show that the 5-year survival rate for NHL has reached approximately 90% [16, 17]. Despite this positive outcome, long-term survivors might experience various adverse health consequences, one of which is the renal late effects. The incidence of late nephrotoxicity varies among studies, depending on the criteria used for diagnosing renal dysfunction and the specific patient populations included in the studies [16, 17]. Additionally, there is a significant lack of studies investigating the late renal effects of TLS [3]. Rasburicase, a recombinant urate oxidase, has been recommended for managing patients at high risk for TLS; however, it was not readily available in our center until recently. In this study, we evaluate the frequency, the characteristics, and the outcomes of TLS. In addition, we assess the long-term renal function in children and adolescent survivors of NHL and TLS who did not receive rasburicase.

## Methods

### Patients and study design

Our study was designed as a single-center retrospective study. Patient data was obtained from patient files and electronic medical records. The study included 107 patients (0-18 years of age) admitted to our hospital between 1998 and 2020 with a diagnosis of NHL, and who received chemotherapy. Patients with a history of kidney disease prior to NHL diagnosis were not eligible for the study. The study was approved by the Sisli Hamidiye Etfal Training and Research Hospital Ethics Committee (Approval number: 2916/ 2020).

### Data collection

Age, gender, weight, height, histopathological subgroup, disease stage, kidney involvement, bone marrow involvement of the patients were recorded. Renal involvement was determined based on radiological features [18]. Staging, was performed according to the St. Jude/Murphy staging system [19].

Serum LDH, urea, creatinine, potassium, phosphorus, calcium and uric acid levels at initial admission, were assessed according to the age adjusted reference range employed by our hospital biochemistry laboratory. Estimated GFR (eGFR) was calculated based on the widely used Schwartz formula in pediatrics, in which the glomerular filtration rate was normalized to body surface area [20, 21]. The highest serum potassium, phosphorus, uric acid, creatinine levels and the lowest serum calcium levels were recorded in the first two weeks after hospitalization. Urine output, the presence of blood pressure elevation, seizures and cardiac arrhythmias, and death were recorded. Hypertension was defined as systolic and/or diastolic pressure values above the 95th percentile, determined by age, gender, and height [22].

The presence of TLS and grading system were defined according to the Cairo Bishop criteria. LTLS is recognized by the presence of two or more abnormalities within three days before starting chemotherapy or during the initial seven days after initiating chemotherapy. These abnormalities consist of increased levels of potassium, phosphate, or uric acid, surpassing the upper limit of normal for age or showing a 25% increase from baseline values. Additionally, a decreased level of calcium, dropping below the lower limit of normal for age or demonstrating a 25% decrease from baseline. CTLS is defined as the presence of one or more of the following in addition to LTLS: serum creatinine levels greater than or equal to 1.5 times the upper limit of normal for age, cardiac arrhythmias/sudden deaths, or seizures [1]. For the development of TLS, cancer type and stage, LDH level, renal involvement, impaired renal function, serum potassium, phosphorus and uric acid level parameters were divided into medium and high-risk groups [23].

The presence and severity of AKI were recorded, with the definition and staging of AKI done according to the Kidney Disease: Improving Global Outcomes (KDIGO) criteria [24]. AKI is defined as either an increase in baseline serum creatinine by >0.3 mg/dL within the first 48 hours, or a 1.5-fold increase in baseline serum creatinine within the first seven days, or a urine output of <0.5 mL/kg/hour for the last six hours. Stage II AKI is defined by a doubling in baseline serum creatinine, or a urine output of <0.5 mL/kg/hour for the last 12 hours. Stage III AKI is defined by a three-fold increase in baseline serum creatinine, or serum creatinine levels exceeding 4 mg/dL, or the initiation of renal replacement therapy, or decrease in eGFR to <35 mL/min/1.73m^2^ in patients under 18 years old, or a urine output of <0.3 mL/kg/hour for the last 24 hours, or anuri for the last 12 hours [24].

The patients were treated with BFM-based protocols. Depending on the risk stratification and the histological subtype, these regimens included various combinations of prednisolone, dexamethasone, methotrexate, vincristine, doxorubicin, daunorubicin, cytarabine, 6-mercaptopurine, 6-thioguanine, ifosfamide, cyclophosphamide, and L-asparaginase [25].

Tumor lysis syndrome prophylaxis was initiated at least 24-48 hours before the start of chemotherapy. The hydration protocol was based on the renal functions of the patients in terms of urine production. All the patients with adequate urine output were treated with hyperhydration regardless of other TLS parameters. The patients diagnosed prior to 2012 were given potassium-free alkaline hydration at a dose of 2500-3000 mL/m2/day. After 2012, prophylactic alkalinization was not performed. Allopurinol was administered at a dosage of 300mg/m2/day. Urate oxidase was not given to any of the patients. After the development of TLS, the serum electrolytes, uric acid, and creatinine levels of the patients were checked every 4-6 hours. Dialysis was administered to patients who had not responded to medical treatment options for TLS [26, 27]. The presence and causes of death during treatment were recorded.

Patients who finished their treatment and remained in remission for at least one year were considered survivors. After completing chemotherapy, their clinical assessments were conducted every 3 months for the initial 2 years post-treatment, followed by evaluations every 6 months for the subsequent 3 years. Afterward, annual assessments were carried out. At the last admission, we recorded the age, height, weight, blood pressure, serum urea, creatinine, electrolyte, and eGFR levels of the survivors. For eGFR calculations, the CKD-EPI (Chronic Kidney Disease-Epidemiology) formula was used for patients aged 18 and above, while the Schwartz formula was applied for those under 18. An eGFR value <90 mL/min/1.73m^2^ was defined as impaired renal function [10, 15, 20, 21].

### Statistical analysis

Statistical analysis for the study was conducted using the statistical software tools NCSS [28] and PASS [29]. During the evaluation of the study data, descriptive statistical methods such as mean, standard deviation, median, frequency, and ratio were employed. Additionally, the Shapiro-Wilk test and boxplot charts were utilized to assess the conformity of variables to the normal distribution. Comparisons between groups with non-normally distributed parameters were performed using the Mann-Whitney U test. To analyze qualitative data, the Chi-square test, Fisher’s exact test, and Fisher-Freeman Halton test were employed. Survival analysis was carried out using Kaplan Meier survival analysis and LogRank test. The significance level was determined at *p*<0.05.

## Results

Our study included 107 pediatric cancer patients, consisting of 21 (19.7%) female and 86 (80.3%) males. The median age of the patients at the time of diagnosis was determined to be 9.8 years (between 1.7-17.4). Burkitt’s lymphoma (BL) was the most common histopathological subgroup (74.8%, 80 patients). The remaining distribution of the patients was as follows: 19 (17.8%) lymphoblastic lymphoma (LL), five (4.6%) diffuse large B-cell lymphoma (DLBCL), and three (2.8%) anaplastic large cell lymphoma (ALCL). The majority of the patients (76.7%) having advanced disease (stage III-IV). There were 12 patients (11.2%) with bone marrow involvement, while there were 11 patients (10.3%) with kidney involvement. We identified two patients with bilateral kidney involvement. At the time of diagnosis, the patients’ median LDH value was 715 U/L. (71-9840). Serum LDH level was found to be less than 500 U/L in 40 patients, between 500-1000 U/L in 25 patients, and greater than 1000 U/L in 42 patients (Table 1). While hyperuricemia was the most common metabolic disorder (7.5%) at the time of admission, hyperphosphatemia was the most common metabolic disorder (18.7%) during treatment. Biochemical parameters in diagnosis and during treatment are summarized in Table 2.

**Table 1:** Patient characteristics of the study population

Age	(Min-Max (Median))	1.7-17.4 (9.8) years
Sex	Male (n (%))	86 (80.3)
	Female (n (%))	21 (19.7)
Histopathological subgroups	Burkitt lymphoma (n (%))	80 (74.8)
	Diffuse large B-cell lymphoma (n (%))	5 (4.6)
	Anaplastic large cell lymphoma (n (%))	3 (2.8)
	Lymphoblastic lymphoma (n (%))	19 (17.8)
Stage	Stage 1 (n (%))	1 (0.9)
	Stage 2 (n (%))	24 (22.4)
	Stage 3 (n (%))	60 (56.1)
	Stage 4 (n (%))	22 (20.6)
Bone marrow involvement	Yes (n (%))	12 (11.2)
	No (n (%))	95 (88.8)
Renal involvement	Yes (n (%))	11 (10.3)
	No (n (%))	96 (89.7)
Lactate dehydrogenase level	<500 U/L (n (%))	40 (37.4)
	500-1000 U/L (n (%))	25 (23.4)
	>1000 U/L (n (%))	42 (39.2)
Lactate dehydrogenase	(Min-Max (Median))	71-9840 (628) U/L
	(Mean±SD)	1037.82±1171.84 U/L
Creatinine	(Min-Max (Median))	0.1-1.17 (0.5) mg/dL
	(Mean±SD)	0.51±0,22 mg/dL
Urea	(Min-Max (Median))	3-56 (20) mg/dL
	(Mean±SD)	21.60±10.35 mg/dL
Estimated glomeruler filtration rate mL/minute/1.73m^2^	>90 mL/minute/1.73m² (n (%))	107 (100)
	<90 mL/minute/1.73m² (n (%))	0 (0)

**Table 2:** Laboratory values of biochemical parameters

		Uric Asid	Potassium	Calcium	Phosporus
		(mg/dL)	(mmol/L)	(mg/dL)	(mg/dL)
At presentation	*(Min-Max (Median)) *	0.5-14.8 (3.45)	2.8-5.5 (4.2)	5.8-11 (9.4)	2.4-13.4 (4.4)
	*(Mean*±*SD)*	4.87±7.79	4.14±0.53	9.36±0.99	4.60±1.39
At presentation serum level	*Increased (n (%))*	8 (7.5)	2 (1.9)	2 (1.9)	7 (6.6)
	*Normal (n (%))*	79 (73.8)	101 (94.4)	98 (91.6)	96 (89.7)
	*Decreased (n (%))*	20 (18.7)	4 (3.7)	7 (6.5)	4 (3.7)
First two weeks after hospitalization	*(Min-Max (Median))*	0.48-14.80 (3.1)	3.7-6.7 (4.6)	5.8-10.4 (8.7)	2.4-25.5 (5.1)
	*(Mean*±*SD)*	3.87±2.69	4.69±0.62	8.49±1.18	5.94±3.40
Serum level in the first two weeks after hospitalization	*Increased (n (%))*	11 (10,3)	5 (4.7)	0 (0)	20 (18,7)
	*Normal (n (%))*	37 (34,6)	102 (95.3)	98 (91.6)	78 (72.9)
	*Decreased (n (%))*	59 (55.1)	0 (0)	9 (8.4)	9 (8.4)

Laboratory TLS was found in 33 (30.8%) of the patients, while CTLS was found in 13 (12.1%). Among the patients experiencing CTLS, respectively; Grade I, II and III CTLS were observed in one patient (0.9%), eight patients (7.4%), and four patients (3.7%) respectively. At the time of admission, four (3.7%) patients experienced spontaneous TLS. All of the TLS patients were found to be in the high-risk group for TLS development. There was a significant correlation between young age, advanced stage, high LDH level at presentation, and LTLS (*p*<0.05). Gender, histopathological subgroup, bone marrow involvement, and kidney involvement had no effect on LTLS (*p*>0.05) (Table 3). The rates of AKI were significantly higher in the group where laboratory TLS was detected (*p*<0.05). CTLS developed at a median of 24 hours (between 0-120) after LTLS. Clinical TLS was more frequently observed in patients with bone marrow involvement and high LDH (*p*<0.05). When age, gender, histopathological subgroup, stage, and renal involvement were compared, there was no statistically significant difference between those with and without CTLS (*p*>0.05) (Table 4). All the patients who experienced AKI were also CTLS patients. Although there was correlation between the grades of AKI and CTLS (*r*$=$0.4606), it was not statistically significant (*p*>0.05).

**Table 3:** Characteristics of children with and without laboratory tumor lysis syndrome

		LTLS		
		Yes (n,%)	No (n,%)	*P value*
Age (year)	n	33	74	*0.018*
	Min-Max (Median)	2.3-15.7 (8.24)	1.7-17.4 (10.49)	
	Mean±SD	8.07±3.6	9.93±3.7	
Gender	Male	24 (72.7)	62 (83.8)	*0.184*
	Female	9 (27.3)	12 (16.2)	
Histopathological subgroups	Burkitt lymphoma	26 (78.8)	54 (73)	*0.765*
	Lymphoblastic lymphoma	4 (12.2)	15 (20.2)	
	DLBCL	2 (6)	3 (4)	
	ALCL	1(3)	2 (2.8)	
Stage	Stage 1-2	2 (6)	23 (31.1)	*0.050*
	Stage 3	23 (69.8)	37 (50.0)	
	Stage 4	8 (24.2)	14 (18.9)	
LDH level at presentation	n	33	74	*0.001*
	Min-Max (median)	71-3969 (1332)	140-9840 (537)	
(U/L)	Mean±SD	*1433.48*±*951.8*	*1433.48*±*951.8*	
Bone marrow involvement		6 (18.2)	6 (8.1)	*0.183*
Renal involvement		3 (9.1)	8 (10.8)	*1.00*

Abbreviations: *ALCL* anaplastic large cell lymphoma, *DLBCL* diffuse large B-cell lymphoma, *LDH* lactate dehydrogenase, *LTLS* laboratory tumor lysis syndrome

**Table 4:** Characteristics of children with and without clinical tumor lysis syndrome

		CTLS		
		Yes (n,%)	No (n,%)	*P value*
Age (year)	*n*	13	94	*0.101*
	Min-Max (Median)	3.52-12.97 (8.38)	1.7-17.41 (10,06)	
	Mean±SD	7.91±3.04	9.56±3.81	
Gender	Male	10 (76.9)	76 (80.9)	*0.716*
	Female	3 (23.1)	18 (19.1)	
Histopathological subgroups	Burkitt lymphoma	12 (92.3)	68 (72.3)	*0.451*
	Lymphoblastic lymphoma	1 (7.7)	18 (19.1)	
	DLBCL	0 (0.0)	5 (5.4)	
	ALCL	0 (0.0)	3 (3.2)	
Stage	Stage 2	1 (7.6)	24 (25.6)	*0.090*
	Stage 3	6 (46.2)	54 (57.4)	
	Stage 4	6 (46.2)	16 (17.0)	
LDH level at presentation	n	13	94	*0.046*
	Min-Max (median)	71-3790 (1056)	140-9840 (617.5)	
	Mean±SD	1477.69±1084.93	984.95±1183.66	
Bone marrow involvement		4 (30.8)	8 (8,5)	*0.038*
Renal involvement		0	11(11.7)	*0.353*

Abbreviations: *ALCL* anaplastic large cell lymphoma, *DLBCL* diffuse large B-cell lymphoma, *LDH* lactate dehydrogenase, *CTLS* clinical tumor lysis syndrome

Acute kidney injury was found in 13 (12.1%) of the patients. Out of these patients, ten (76.9%) were male, and three (23.1%) were female. The most prevalent tumor subtype was BL, observed in 92.3% of cases (12 patients), followed by LL at 7.7% (one patient). None of the patients diagnosed with DLBCL and ALCL experienced AKI. Among those who did experience AKI, six (46.2%) were at Stage IV, six (46.2%) were at Stage III, and one (7.6%) was at Stage II. Notably, none of the NHL patients at Stage I exhibited AKI. Stage II AKI in three (2.8%) patients, and Stage III AKI in nine (8.4%) patients. There was no evidence of renal involvement in patients with AKI. When renal involvement was compared between those with and without AKI, no statistically significant difference was observed (*p*>0.05). Hyperphosphatemia (84.6%, 11 patients) was the most common metabolic disorder associated with AKI, followed by hyperuricemia (46.1%, 6 patients). Hyperuricemia and hyperphosphatemia were significantly higher in those who experienced AKI compared to those who did not (*p*<0.05). Dialysis was performed on five (4.6%) patients due to hyperphosphatemia and hyperuricemia that did not respond to medical treatment. Two (1.9%) patients developed cardiac arrhythmia, one (0.9%) patient had seizures due to hypocalcemia, and one (0.9%) patient developed hypertension.

**Fig. 1:**
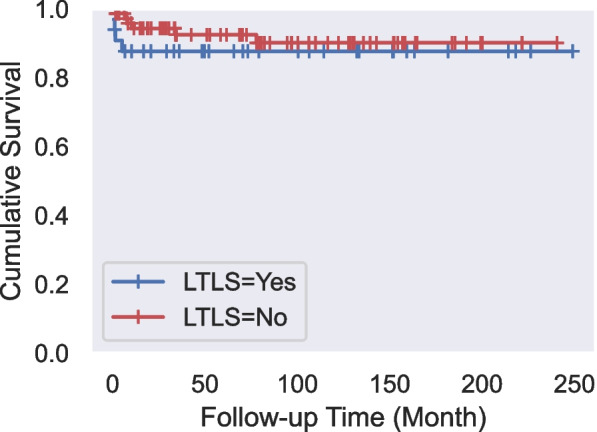
Survival analysis for laboratory tumor lysis syndrome (LTLS)

**Fig. 2:**
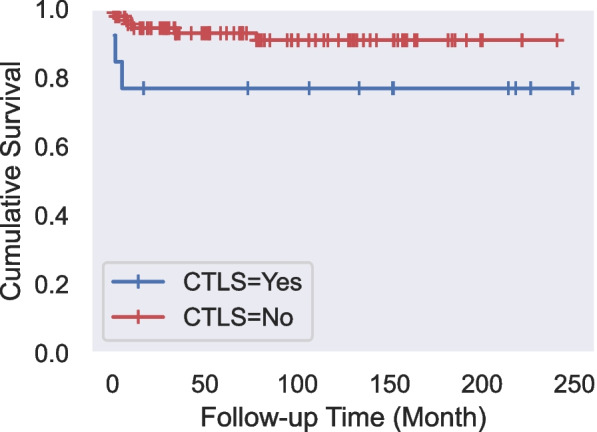
Survival analysis for clinical tumor lysis syndrome (CTLS)

The median follow-up period of patients was 6.2 years (1 year-19.3 years) (mean 6.9 years). During the follow-up period, the disease recurred in two patients, and one patient developed secondary acute lymphoblastic leukemia (ALL). Mortality was observed in 10 patients (9.3%) due to sepsis, pneumonia, or respiratory failure. TLS did not cause any deaths. The last death was seen at the 56th month; the cumulative survival rate in this month is 88.7%, with a standard error of 3.5%. The survival times for cases with and without LTLS were 192.35±6.73 months and 209.11±9.81 months, respectively, with a mortality rate of 13.4% and 8.3% (Fig. 1). When the survival rates according to LTLS were evaluated with the LogRank test, there was no statistically significant difference between the survival rates (p=0.193; p>0.05). The survival times for cases with and without CTLS were 165.50±26.55 months and 219.90±7.51 months, respectively, with a mortality rate of 23.1% and 7.8% (Fig. 2). When the survival rates according to CTLS were evaluated with the LogRank test, there was no statistically significant difference between the survival rates (p=0.073; p>0.05).

Serum urea and creatinine levels were within normal limits for age in all patients at the last follow-up. All patients had eGFR value of $\ge$90 mL/min/1.73m2. When the glomerular filtration rate values of the patients at the first and last admissions were compared after an average of 6.9 years, a mean decrease of 10 mL/min/1.73 m2 was found (p=0.0693). In the long term, only one (0.9%) patient developed Stage I hypertension due to cardiological late effects.

## Discussion

Non-Hodgkin lymphoma stands as one of the prevalent cancer types among children [30]. TLS poses a medical emergency with a mortality risk. Nevertheless, there is a scarcity of literature on the delayed renal functions in affected children [3]. Our retrospective study, conducted at a single center over a span of more than two decades, underscores the significance of assessing renal functions in long-term survivors. Our research provides a comprehensive examination of the frequency of TLS, its risk factors, laboratory and clinical features, and its impact on the long-term renal functions in pediatric NHL.

Male gender was more prevalent in our study, as previously described in the literature. The most common histopathological subgroup is BL, and the majority of patients presented with advanced disease associated with large tumors, Stages III and IV according to the St. Jude/Murphy staging criteria [19, 30]. The fact that most of the patients were diagnosed at the advanced stage could be attributed to delayed admission to the hospital caused by the socioeconomic conditions of the period (our study covers a period of 22 years).

The incidence of TLS varies depending on many reasons including the presence of tumors with a high proliferation rate, heterogeneity of the patient population and treatment regimens, differences in TLS prophylaxis, and the lack of a universal TLS definition [9, 31]. In a multicenter study of 1791 pediatric patients with advanced NHL and B-cell ALL in 2003, Wossman et al. detected TLS in 4.4% of the patients. TLS was most frequently observed in patients with BL (14.9%) and B-ALL (26.4%) [8]. In the study conducted by Sevinir et al. in which 327 patients with NHL and ALL were retrospectively analysed, the incidence of TLS was found to be 15.9% in NHL [27]. In our study, LTLS was observed at a higher rate (30.8%) compared to the literature. The fact that the majority of our study group consisted of advanced-stage BL patients with increased tumor burden may have attributed to a higher frequency of TLS in our study.

Cairo et al. created a risk classification to predict patients who are likely to develop TLS, which includes both solid tumors and hematological malignancies [23]. According to this classification, in our study, all patients who underwent TLS were classified in the high-risk group. The absence of TLS in the intermediate risk group indicates to that Cairo et al.’s classification has a high predictive value.

Tumor lysis syndrome risk factors include advanced stage, high LDH levels, bone marrow involvement, and the presence of a highly proliferating tumor [27]. Our study’s findings were consistent with the literature. However, we identified lower age as another risk factor for TLS. EGFR and tubular function increase with age from birth [14]. As a result, younger children may be more prone to dehydration, ischemia, and AKI than adolescents. However, we did not find a correlation between lower age and CTLS. The elevated metabolic rate in children increases caloric expenditure and higher fluid requirements [32]. Moreover, children, especially infants, exhibit a significantly higher body surface area-to-weight ratio, resulting in more water loss through the skin compared to adults. Additionally, they have higher respiratory rates, contributing to greater insensible losses from the respiratory tract. All these factors render younger children more susceptible to dehydration. Consequently, the frequency of fluid loss might be more common in the younger group, potentially mitigated with prompt rehydration in our center, preventing progression to CTLS. However, given the retrospective nature of our study, parameters such as dehydration and prior nephrotoxic agent exposures might not have been evenly distributed among groups, limiting a detailed evaluation of these parameters. Furthermore, in our study, the number of patients with CTLS is relatively smaller than those with LTLS, reducing the significance of statistical comparisons.

Renal involvement in lymphoma was not found to be a risk factor for TLS in our study, similar to previously reported in the literature [33]. In our study, eGFR values of patients with kidney involvement were found to be within normal range at admission, with no accompanying obstructive nephropathy. This suggests that renal involvement does not contribute to LTLS development.

It is well-known that the most common metabolic disorder associated with TLS is hyperuricemia [33–36]. In our study, hyperuricemia was found to be the most common metabolic disorder at admission, consistent with the literature. Hyperphosphatemia was the most common metabolic disorder in the first two weeks after starting chemotherapy. This shift in metabolic disturbance is understandable given that early administration of hyperhydration and allopurinol prevents uric acid accumulation while medical measures, with the exception of dialysis, have limited activity against phosphorus elimination. Oral agents only bind phosphorus absorbed from the gastrointestinal tract, with insufficient efficacy and low tolerance but the main source of phosphorus in TLS is malignant cells [4, 27, 37]. The frequency of hypocalcemia observed during treatment was found to be higher than that observed during diagnosis in our study. Excess phosphorus binds calcium in soft tissues, including the kidney, and causes the precipitation of calcium phosphate crystals, resulting in hypocalcemia [4, 37]. In our study, hypocalcemia observed during treatment is thought to be secondary to hyperphosphatemia.

Acute kidney injury is an important complication of TLS that can cause mortality. In our study, the rates of AKI were found to be significantly higher in patients who developed TLS compared to those who did not (*p*<0.05). As in TLS, the heterogeneity of the patient population and treatment regimens and the incidence of AKI vary according to cancer type [10, 15, 38]. In a study conducted by Park et al., [10] AKI was defined according to the criteria of KDIGO in 1868 pediatric cancer (ALL, AML, NHL, neuroblastoma, Wilms tumor, brain tumor, other tumors) patients in 2019. According to this study, AKI developed in the first year of cancer treatment in 983 (52.6%) patients. In our study, AKI was defined in 13 (12.1%) of our patients according to the KDIGO criteria.

In our study, out of 13 patients experiencing AKI, 12 (92.3%) were diagnosed with BL, and 12 (92.3%) exhibited Stage III-IV tumors. As mentioned earlier, BL is one of the hematologic malignancies demonstrating a high proliferation rate. Increased tumor burden (Stage/LDH) and rapid proliferation lead to more tumor cell necrosis, resulting in the release of more anionic and cationic breakdown products of nucleic acids into circulation. This condition can surpass the renal tubules’ excretory capacity, potentially exacerbating the development of AKI.

In a multicenter prospective study conducted by Darmon et al. On a total of 153 adult patients diagnosed with acute myeloid leukemia, ALL, and NHL, it was found that 1-mmol increase in serum phosphate level caused a fivefold increase in the risk of CTLS (together AKI) [38]. In our study, the most common electrolyte disorder associated with AKI was hyperphosphatemia (78.5%). With the advent of hypouricemic agents, it is possible that the hyperphosphatemia has begun to emerge as the primary metabolic indicator related to LTLS and AKI.

Tumor lysis syndrome has been linked to an increase in mortality in studies [39]. A decreasing survival trend with CTLS was also observed in our study. CTLS patients have a significant tumor burden (advanced disease). As a result, they may be considered high-risk patients in terms of mortality.

Childhood cancer survival has now risen to 80% [40]. This success, however, comes at a cost, and the long-term effects of pediatric cancer treatment are a problem in adulthood. Nephrotoxicity is well documented in childhood cancer survivors and the possible causes include infections, dehydration, TLS, surgery and radiation therapy as well as nephrotoxic agents such as antineoplastic drugs, certain antibiotics and contrast agents. All these insults can cause AKI which increase the future risk of chronic kidney disease [10, 12, 13, 15]. In our study, long-term renal function loss was not detected in the survivors. The decline in renal function may become more pronounced as the follow-up period lengthens. The addition of clinical conditions seen in adulthood, such as cardiovascular disease, dyslipidemia, insulin resistance, and hypertension, may result in a decrease in kidney function in the future. Therefore patients’ kidney functions should be monitored in the long term for the development of progressive kidney disease.

Our study has certain limitations. Due to its retrospective nature, there are limitations in retrospectively reviewing some medical data related to patients. Another limitation of our study is its single-center nature, resulting in a relatively small patient population.

In conclusion, Our study evaluated the prevalence and clinical/laboratory characteristics of TLS in children with NHL as well as assessed long-term renal functions of survivors. The main result was zero TLS-related mortality with conventional approach without the use of rasbirucase and preserved renal functions in survivors with a history of TLS.

## Data Availability

The datasets used and/or analysed during the current study available from the corresponding author on reasonable request.

## Abbreviations

AKI: Acute kidney injury
ALCL: Anaplastic large cell lymphoma
ALL: Acute lymphoblastic leukemia
BL: Burkitt’s lymphoma
CKD-EPI: Chronic Kidney Disease-Epidemiology
CTLS: Clinical tumor lysis syndrome
DLBCL: Diffuse large B-cell lymphoma
eGFR: Estimated glomerular filtration rate
KDIGO: Kidney Disease: Improving Global Outcomes
LDH: Lactate dehydrogenase
LL: Lymphoblastic lymphoma
LTLS: Laboratory tumor lysis syndrome
NHL: Non-Hodgkin lymphoma
TLS: Tumor lysis syndrome
